# Penetration of Three Endodontic Sealers in Simulated Lateral Canals during the Lateral Condensation Technique: An In Vitro Study

**DOI:** 10.1155/2022/2686247

**Published:** 2022-11-16

**Authors:** Mohamed El Sayed

**Affiliations:** ^1^Restorative Dentistry Department, College of Dentistry, Ajman University, Ajman, UAE; ^2^Endodontic Department, Faculty of Dentistry, Mansoura University, Mansoura, Egypt

## Abstract

**Objective:**

This study aimed to compare the penetration depths of AH Plus, BioRoot RCS, and GuttaFlow 2 into simulated lateral canals when used with the cold gutta-percha lateral compaction technique.

**Materials and Methods:**

Twelve resin training blocks (4 canals perch each resin block) were used. Each primary artificial canal had two lateral canals (apical and coronal). The main canals were instrumented with WaveOne Gold and irrigated with distilled water. The resin blocks were divided into three groups (*N* = 4 each/16 artificial canals), according to the type of root canal sealer; Group I: AH Plus, Group II: BioRoot RCS, and Group III: GuttaFlow 2. All canals were obturated with the cold lateral condensation technique. The linear extension of each endodontic sealer into the apical and coronal lateral canals was measured using a digital stereomicroscope and measuring software. Data were statistically analyzed using a one-way analysis of variance. The percentages of filling of the lateral canals were calculated and statistically compared using the Mann–Whitney test.

**Results:**

The experimental sealers exhibited variable penetration depths into the lateral canals. All sealers showed significantly better penetration ability into the apical lateral canals than the coronal lateral canals (*P* < 0.05). AH Plus (3.184 ± 0.012 mm/99.5%) and GuttaFlow 2 (3.176 ± 0.017 mm/99.25%) were significantly better than BioRoot RCS (3.096 ± 0.026 mm/96.75%) in filling the apical lateral canals (*P* < 0.05). BioRoot RCS was the best sealer to fill coronal lateral canals (3.322 ± 0.085 mm/83.05%).

**Conclusion:**

During the lateral condensation technique, the filling of the lateral canals is affected by the type of root canal sealer and the location of the lateral canals All the sealers tested have a good ability to fill the apical lateral, while BioRoot RCS was effective in filling both the coronal and apical lateral canals.

## 1. Introduction

Infection of the root canal system is considered the main cause of periapical lesions [1]. Inadequately filled areas in a well-prepared root canal system can be a source of microbial growth, as 58% of treatment failures were reported to be due to insufficient obturation [2]. Therefore, a three-dimensional filling of the canal space with an inert biocompatible material is required to prevent bacterial leakage and reinfection of the root canal system [3].

Total filling of the root canal system is a clinical challenge due to its inherent morphological complexities [4]. The root canal system may be complicated by the presence of several ramifications, such as lateral and accessory canals, which harbor some pulp tissues and microorganisms [5]. The incidence of lateral canals is relatively high, ranging from 27.4 to 99% [6], and may be considered a cause of failure after endodontic treatment if they are not properly cleaned and sealed [7]. There is a strong correlation between infection inside root canals and the presence of apical periodontitis [7]. Furthermore, some studies have reported endodontic success after the obturation of lateral canals [8, 9]. Consequently, root canal filling procedures should include the filling of the main root canal, as well as the lateral and accessory canals [10].

Despite the development of numerous obturation techniques, the cold lateral condensation technique is considered the most common obturation technique, as it is simple and does not require sophisticated equipment, and is easy to learn [11]. Additionally, several authors showed that the lateral condensation technique has a sealing ability similar to other newly developed obturation techniques [12].

Root canal sealers play an important role in the success of any obturation technique. Therefore, it is important to use a sealer with good sealing, antibacterial, and flow properties, as well as radiopacity, dimensional stability, and low cytotoxicity [13]. To this day, there is no product that combines all the ideal properties, which may explain the need for the continuous development of new endodontic sealers [14]. Currently, there are various types of contemporary endodontic sealers on the market, such as AH Plus (Dentsply De Trey GmbH, Konstanz, Germany), GuttaFlow 2 (Coltène/Whaledent, Altstätten, Switzerland), and more recently the BioRoot RCS (Septodont, Saint-Maur-des-Fosses, France).

AH Plus (DeTrey Dentsply GmbH, Konstanz, Germany) is an epoxy resin-based endodontic sealer that exhibits excellent physical properties and bond strength with dentin, [13, 15]. One of the advanced materials on the market is GuttaFlow (Coltène Whaledent, Altstatten, Switzerland), which is based on silicone-based sealer and gutta-percha powder. More recently, this product has been modified, giving rise to GuttaFlow 2 which has good physicochemical properties, low cytotoxicity, and good adhesiveness to dentin [16–18].

Recently, bioceramic root canal sealers have been released on the market and became popular among endodontists due to their excellent physical and biological properties. These materials showed auspicious properties of radiopacity, flow, osteoconductivity, alkaline pH, low cytotoxicity and genotoxicity, and adequate antibacterial effectiveness [19]. BioRoot RCS (Septodont) is a type of bioceramic sealer composed of tricalcium silicate, zirconium oxide, and calcium chloride. It is indicated for permanent root canal filling in combination with gutta-percha cones and is suitable for use in single-cone or cold lateral condensation techniques [20].

Variable methods have been used to study the filling of lateral canals. Goldberg et al. [21] used natural human teeth after drilling simulated lateral canals and then examined the filling of the lateral canals radiographically. Venturi et al. [22] used the same previous method but examined the filling of lateral canals using the clearing technique. Dulac et al. [23] used resin blocks after drilling simulated lateral canals. Venturi et al. [24] used Thermafil training resin blocks to study the penetration of root canal sealers into the lateral canals.

Currently, limited information on the penetration of BioRoot RCS and GuttaFlow 2 into lateral root canals is available when used with the lateral condensation technique. Therefore, the present study aimed to evaluate the ability of AH Plus, BioRoot RCS, and GuttaFlow 2 to fill artificial lateral canals in two different locations, when the lateral condensation technique was used. The null hypothesis tested was that there are no significant differences between the penetration capacities of experimental sealers into artificial lateral canals at different locations when used with the cold lateral condensation technique.

## 2. Materials and Methods

The Research Ethics Committee of Ajman University, UAE under protocol number UGD-H-18-12-24-44, approved the present study.

### 2.1. Thermafil Resin Training Blocks

**Figure 1:**
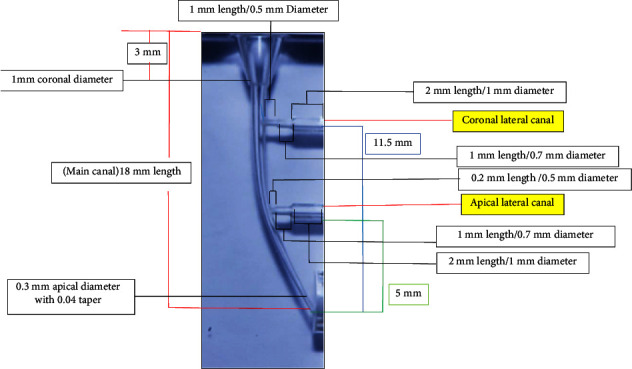
Dimensions of the main canal and lateral canals within the Thermafil resin training block.

Thermafil training blocks (Dentsply Maillefer) were used in the current study (Figure 1). Each resin block had four primary canals of 18 mm in length and 25° curvature. Each main canal had two lateral canals located at 5 mm (apical lateral canal) and 11.5 mm (coronal lateral canal) from its apical end. Each lateral canal had three successive cylinders (inner, middle, and external) of different lengths and diameters. The overall length of the apical lateral canals was 3.2 mm (inner length: 0.2 mm, middle length: 1 mm; coronal length: 2 mm), while the length of the coronal lateral canals was 4 mm (inner: 1 mm, middle: 1 mm; coronal: 2 mm). The diameters of the internal, middle, and external cylinders in both lateral canals were 0.5, 0.7, and 1 mm, respectively. The diameter of the coronal orifice of each primary canal, 3 mm from the surface of the resin block, was 1 mm and its apical end diameter was 0.3 mm with a 4% taper. The dimensions of the main and lateral canals mentioned above were according to Venturi et al. [24] and were confirmed in the current study using a digital microscope and measurement software.

### 2.2. Samples Size Calculation

An experimental design of repeated measures was performed. Sample size estimation was performed a priori using G^*∗*^Power 3.1.9.6 (Universität Kiel, Kiel, Germany) [25], assuming that a standardized effect size of 0.7276 should be detected by repeated measures ANOVA at 95% power and with a two-tailed probability of alpha type error of 0.05. Finally, a sample size of 16 artificial canals was selected for each group in the present study (4 resin blocks), resulting in a total of 16 artificial canals with 32 artificial lateral canals.

### 2.3. Canal Instrumentation

Canal instrumentation procedures were performed under an endodontic microscope at 16x magnification (Carl Zeiss Microscopy, LLC). The patency of all main and lateral canals was verified using a #20 stainless steel K-file (Dentsply, Maillefer). The working length (WL) was established at 1 mm short of the standardized length of each main canal (WL = 17 mm). All canals were prepared with WaveOne Gold reciprocating endo file size 45/0.05 (Dentsply, Maillefer Sirona) according to the manufacturer's instructions. EDTA gel (Glyde File Prep, Dentsply, Maillefer, Switzerland) was used as a lubricant during instrumentation procedures. Three cutting cycles were used at three working lengths of 5.5 mm in length. After each cutting cycle, the file was removed, and its flutes were cleaned from any resin debris and inspected for any visible distortion or unwinding. The root canals were irrigated with 5 ml of distilled water after each preparation cycle using a side-vented needle (Max-I-Probe, Dentsply). After the instrumentation was completed, the patency of the main and lateral canals was again verified with a #20 K file. According to previous studies, each WaveOne gold file was used to prepare four canals and then discarded, to ensure its cutting efficiency [26, 27].

### 2.4. Samples Grouping

After finishing the preparation of all artificial canals, the thermal fill training blocks (*N* = 12 resin block) were divided, according to the type of root canal sealers, into three groups of 4 resin blocks each (16 prepared canals/32 lateral canals) Group I: AH Plus, Group II: BioRoot RCS, and Group III: GuttaFlow 2. The composition of each experimental sealer is shown in Table 1.

### 2.5. Canal Obturation

Before starting the obturation phase, each acrylic block was covered with a layer of heavy body impression material (ZetaPlus, 3M ESPE, Saint Paul, USA) to simulate the periodontium. The prepared canals were dried with Wave One Gold paper points (large size). The canals of the Bioroot RCS group were dampened using a moistened paper point to ensure the setting of this hydrophilic sealer. The selected root canal sealer (AH Plus; Group I, BioRoot RCS; Group II; GuttaFlow 2; Group III) was mixed according to the manufacturer's instructions and applied to the prepared resin canal using a #30 Lentulo spiral rotated at 300 rpm and 3 mm shorter than the working length. The tip of preselected master gutta-percha cone size 45/0.02 was lightly coated with sealers and slowly inserted into the canal until it reached the full working length. Lateral condensation was performed using a premeasured size C finger spreader (D1 diameter 0.3 mm, 0.04 taper) (Dentsply, Maillefer) and standardized gutta-percha cones of size 25/0.02. Accessory cones were added and lateral condensation was continued until the spreader could not penetrate more than 3 mm from the surface of the acrylic block. The excess gutta-percha was seared using a hot instrument and lightly vertically compacted. All obturated samples were stored at 37°C and 100% humidity in an incubator for one week to allow the sealers to be completely set.

All instrumentation and obturation procedures were done by the author who is a specialist in endodontics.

### 2.6. Measurement of Sealer Penetration into Lateral Canals

**Figure 2:**
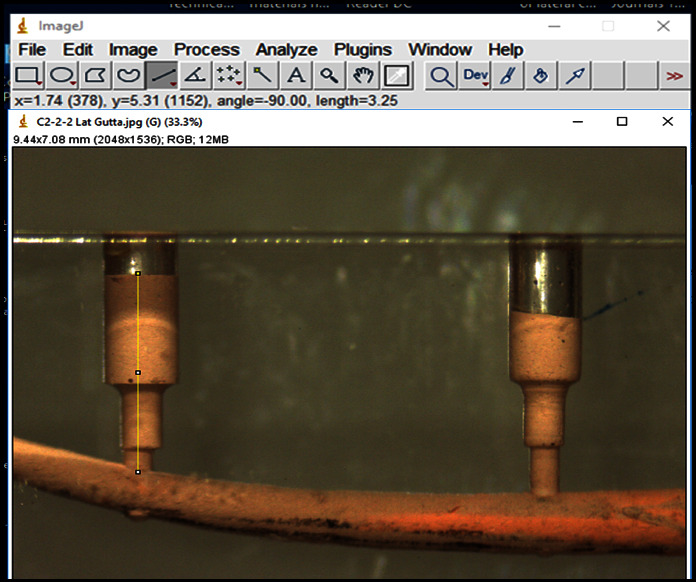
Measuring the sealer penetration depth (mm) into coronal and apical lateral canals using imageJ software.

All filled canals were inspected with a digital stereomicroscope (Leica EZ4W, Germany) at 12.5x magnification and photographed from the same aspect at the outer margin of the resin block to standardize the evaluation (Figure 2). Photographic images showing the root canal fillings in each specimen were imported into Image Tool software (ImageJ software, National Institutes of Health, Bethesda, USA). After calibrating the software, the length of sealer penetration into each lateral canal was measured in mm and also expressed as a percentage of linear extension (length of the filled portion of the lateral canal divided by its entire length). Two independent examiners, previously calibrated and blinded to the study, measured the sealer penetration into the artificial lateral canals at two different time intervals according to the assessment criteria prescribed previously.

### 2.7. Statistical Analysis

Intraexaminerand interexaminer reliability for sealer penetration measurement was verified by the kappa test. Data were analyzed using IBM SPSS version 20 (IBM Corporation 1 New Orchard Road Armonk, New York, USA). After verifying the normality of the distribution of the results using the Kolmogorov–Smirnov test, the ANOVA followed by the Games–Howell test or Tuckey post hoc test was used to compare the groups for each lateral canal. The mean percentages of penetration of each sealer into the apical and coronal lateral canals were also compared using the Mann–Whitney test. The level of statistical significance was set at *P* < 0.05.

## 3. Results

The level of interexaminer agreement was very high, attaining a kappa value of 0.92 [28]. The means and standard deviations of the penetration depths of sealers in the lateral canals are presented in Table 2. Furthermore, the percentages of filling of the lateral canals with experimental sealers are presented in Table 3. All experimental sealers showed variable penetration depths into the apical and coronal lateral canals. When the penetration depths of the experimental sealers into the apical and coronal lateral canals were compared, the one-way ANOVA test showed highly significant differences between the groups (*P* < 0.05). Regarding lateral apical canals, AH Plus (3.184 ± 0.012 mm/99.5%) and GuttaFlow 2 (3.176 ± 0.017 mm/99.25%) showed better penetration depths into the apical lateral canals than BioRoot RCS (3.176 ± 0.017 mm/96.75%). Regarding the coronal lateral canals, BioRoot RCS showed the highest penetration ability (3.322 ± 0.085 mm/83.05%). Based on the results of the Mann–Whitney test (Table 3), the apical lateral canals showed significantly higher percentages of sealer filling than the coronal lateral canals (*P* < 0.05).

## 4. Discussion

The filling of the lateral canals is clinically important for any obturation technique to overcome the possible bacterial growth and reinfection of the root canal system [10, 18]. Several studies have reported endodontic success after lateral canal filling; different filling techniques were proposed to achieve better obturation of these canals [11]. Therefore, the present study aimed to compare the penetration ability of three contemporary endodontic sealers into simulated lateral canals when used with the lateral condensation technique.

In the current study, ready-made training blocks were used, in which the dimensions of the main and lateral canals are standardized. Therefore, the variations associated with the instrumentation and filling procedures of the main canals were also standardized [23]. The only drawback of these training blocks is that the surface texture of the epoxy resin blocks is not similar to the natural tooth structure, which may affect the flow of the endodontic sealer. Additionally, the internal diameters of the simulated lateral canals (500, 700, and 1000 *μ*m) used in this study were larger than the natural diameters of the natural lateral canals [22]. However, the filling of large lateral canals may indicate the ability of root canal sealers to fill narrow canals.

To reduce variability in instrumentation procedures, all simulated canals were prepared and obturated by one operator. To standardize the preparation size of the canals, WaveOne Gold file size 45/0.05 was used to prepare the main canals, which have an initial size of 0.30 mm and a taper of 4%. During instrumentation, EDTA gel was used to decrease the friction between the file and canals, and collect the resin debris to be easily removed during the irrigation. Irrigation was done with distilled water, as artificial resin canals were used. The terminal ends of the main and lateral canals were blocked with a rubber base impression material prior to filling the main canal according to the idea described by Almeida et al. [29]. This procedure allowed the sealer to be confined after it had flowed through the lateral canals in an attempt to simulate the role of the periodontal ligament.

The combination of gutta-percha with a suitable endodontic sealer is usually used for root canal filling. The filling of the lateral canals may be affected by the obturation technique [15] and the physical-chemical properties of the endodontic sealer [17]. Cold lateral condensation was selected in the current study, as it is still the technique most used clinically, as it is a relatively simple technique and it can achieve a good sealing ability that was similar to other obturation techniques [11, 12]. In addition, the main goal of this study was to evaluate sealers and not filling techniques in terms of filling lateral canals.

The quality of endodontic sealer plays an important role in the filling of the ramifications and lateral canals. In the present study, three types of sealers were selected, AH Plus, BioRoot RCS, and GuttaFlow 2, based on their excellent physical properties and clinical performance [16]. The selected root canal sealers were mixed according to the manufacturer's instructions and placed in the prepared canals using a Lentulo spiral. Guinesi et al. stated that the use of the Lentulo spiral is a crucial method to place sealer within the root canals when the single-cone obturation technique is used [30].

An adequate flow of endodontic sealers is a fundamental physical property that allows them to fill small spaces, accessory canals, and spaces between the master and accessory cones [16]. Karabucak et al. concluded that the flow of filling material into the lateral canals is a function of the viscoelastic properties of the filling material rather than the mechanical properties of the delivery systems [31].

The pseudoplastic and thixotropic properties of endodontic sealers affect their flowability by decreasing their viscosity when subjected to pressure or stress [16]. According to Hubbe et al., the thixotropic material transforms its internal structure under constant shear stress, which promotes the alteration of the flow speed, accounting for the abrupt flow, after a certain time [32]. Pseudoplastic material exhibits the same changes as a result of increasing the rate of shear stress [16]. Thixotropic materials have a higher viscosity when moved at a slow speed and a lower viscosity when moved at a higher speed [16].

GuttaFlow 2 and AH Plus are thixotropic materials for which their viscosity decreases and their flow increases under constant shear stress [33]. On the other hand, a bioceramic-based sealer is pseudoplastic material that shows the same behavior as thixotropic materials but with an increasing rate of shear stress [16]. Some authors found that bioceramic sealers have higher flowability compared to GuttaFlow and AH Plus [16]. Also, it has been confirmed that GuttaFlow 2 has a better flow than AH Plus [33]. Regarding BioRoot RCS, some authors found that it has a larger film thickness and lower flow than AH Plus [34].

In the literature, there are few studies on the penetration capacity of BioRoot RCS and GuttaFlow 2 into lateral canals when used with the lateral condensation technique. The present results showed that the filling of the lateral canals was affected by the type of endodontic sealers and the location of the lateral canals. Therefore, the null hypothesis was rejected.

The results of the present study indicated that the lateral apical canals had high percentages of filling with all experimental sealers. This finding is clinically significant because it is well-known that there is a greater percentage of lateral canals in the apical third of the root. This can be clarified because all sealers have thixotropic and pseudoplastic properties [16, 33]. Under constant or increased shear stresses during lateral compaction procedures, the viscosity of the sealers decreases, and their flow increases [16, 33]. However, the penetration ability BioRoot RCS in the apical lateral canals (3.096 mm/96.75%) was slightly lower than that of AH Plus (3.184 mm/99.5%) and GuttaFlow 2 (3.176 mm/99.25%). The cause of this may be due to its high viscosity when subjected to more shear stresses that relatively decrease its flow [16]. Moreover, some authors found that the BioRoot RCS has more film thickness and lower flow than that of the AH Plus [34]. The present results are in conflict with Venturi et al. [22] and Goldberg et al. [4] who concluded that the lateral canals localized in the apical third of the root were more difficult to fill. The causes of this conflict may be due to the difference in materials and methods. The findings of GuttaFlow 2 are consistent with the results of Zielinski et al. who showed that GuttaFlow can fill all depressions and groves at the apical one-third of the root canal [35].

The results of this study indicated that all tested sealers showed lower percentages of penetration into the coronal lateral canals than into the apical lateral canals. During the cold lateral compaction of the gutta-percha cones, the generated shear stress is not continuous but instant. This force is dissipated both by decreasing the pressure on the walls with increasing canal taper and by losing the mass of the cement, resulting in reduced flow and incomplete filling of the lateral canals [36]. Despite the low flow of BioRoot RCS [34], it showed better penetration ability into coronal lateral canals (3.32 mm/83%) than AH Plus (2.84 mm/71%) and GuttaFlow 2 (2.69 mm/67.28%). This may be due to the location of these lateral canals in the straight portion of the canal and the pushing of the sealer coronally to penetrate the lateral canal. Additionally, the existing results are somewhat in agreement with the results of Teixeira et al. who showed that the GuttaFlow 2 sealer had less penetration ability into the apical lateral canals (secondary canals) when used with the cold lateral compaction technique [37]. However, the authors showed that AH Plus had better penetration ability into apical lateral canals. The reason for this incongruity was the difference in methodology. Those authors manually prepared the root canals of natural teeth using a step-back technique, and the two coronal and apical artificial lateral canals of smaller diameters were mechanically prepared within the roots.

The presented results cannot be straightforwardly applied clinically, because the penetration of filling materials could be hindered clinically by some factors, such as the diameter of the lateral canals and the lack of patency due to the presence of remnants of the pulp tissue, or dentin debris [7]. However, the results can be used as an indicator of which root canal sealer has a better ability to penetrate the lateral canals when used with cold lateral condensation techniques. The proper irrigation of the root canal system is the only way to clean the lateral and accessory canals and allow sealers to fill them [7]. Kanumuru et al. in their study found that negative pressure irrigation (EndoVac system) and passive ultrasonic irrigation (PUI) promoted better cleaning of the main and simulated lateral canals than the conventional manual irrigation technique [38].

In addition to the penetration ability of root canal sealers into the lateral canals, other important characteristics must be respected during the selection of root canal sealers such as tissue biocompatibility, antimicrobial property, and sealing ability. More studies may be required to study the penetration ability of the investigated sealers into the lateral canals when used with other obturation techniques such as the matched-tapersingle-cone technique.

Based on the results of the current study, the penetration ability of root canal sealers in lateral canals is affected by the type of root canal sealer and the location of the lateral canals. All sealers used with the lateral condensation technique showed significantly better filling for the apical lateral canals than the coronal lateral canals. AH plus and GuttaFlow 2 showed better penetration into the apical lateral canals than BioRoot RCS. BioRoot RCS showed a higher penetration ability into the coronal lateral canals than AH Plus and GuttaFlow 2. BioRoot RCS could be a suitable sealer to be used with cold lateral compaction, which can adequately fill the apical and coronal lateral canals simultaneously.

## 5. Conclusions

Based on the results of the current study, the penetration ability of root canal sealers in lateral canals is affected by the type of root canal sealer and the location of the lateral canals. All tested sealers had a better ability to fill the apical lateral canals than the coronal lateral canals. BioRoot RCS showed the best penetration ability into both apical and coronal lateral canals and could be considered a suitable sealer to be used with cold lateral compaction.

### 5.1. Significant of the Study

Filling the lateral canals after proper cleaning with an efficient irrigation protocol is clinically important. The lateral canals can be filled with endodontic sealers during the lateral compaction technique. The type of sealer plays a critical role in filling the lateral and accessory canals. Most of the lateral canals are confined in the apical area of the roots., but some of them may be found coronally. All sealers tested showed good penetration in the apically positioned lateral canals when used with the lateral compaction technique. However, BioRoot RCS was the only sealer that could fill the coronal and apical lateral canals. Meanwhile, this sealer has excellent sealability and biocompatibility as well as antibacterial properties.

## Figures and Tables

**Table 1:** Composition of the experimental root canal sealers.

Endodontic sealers	Manufacturer	Composition	Lot. no.
AH plus	Dentsply DeTrey GmbH, Konstanz, Germany	Epoxy resin, calcium tungstate, zirconium dioxide, aerosol, iron oxide, adamantine amine, N, N′-dibenzyl-5-oxa-nonandiamine-1,9, coloring, TCD-Diamine, silicone oil	1807000515
BioRoot RCS	Septodont, Saint-Maur-des-Fosses, France	Powder: tricalcium silicate, zirconium dioxide. Liquid: water, calcium chloride, polycarboxylate, and povidone	B22432
GuttaFlow 2	Coltène/Whaledent, Altstätten, Switzerland	Gutta-percha powder particles, polydimethylsiloxane, platinum catalyst, zirconium dioxide, microsilver particles, coloring	J01342

**Table 2:** Comparison between experimental sealers regarding their penetration into the apical and coronal lateral canals.

Groups	Mean values ± SD (mm) of sealer penetration	
	Apical lateral canals (total length: 3.2 mm)	Coronal lateral canals (total length: 4 mm)
Group I (AH Plus)	3.184 ± 0.012^A^^*∗*^	2.84 ± 0.056^A^^*∗∗*^
Group II (BioRoot RCS)	3.096 ± 0.026^B^	3.322 ± 0.085^B^
Group III (GuttaFlow 2)	3.176 ± 0.017^A^	2.691 ± 0.063^C^
ANOVA (*P* value)	0.000	0.000

*∗*Games–Howell post hoc test; ^*∗∗*^Tukey test: means with different manuscript letters within each column are significantly different at *P* < 0.05.

**Table 3:** Comparing the percentages of filling of apical and coronal lateral canals with each experimental endodontic sealer.

Groups	Percentages of filled lateral canals (%) ± SD		
	AH Plus	BioRoot RCS	GuttaFlow 2
Apical lateral canals	99.5 ± 0.360^A^*∗*	96.75 ± 0.798^A^	99.25 ± 0.534^A^
Coronal lateral canals	71 ± 1.392^B^	83.05 ± 2.116^B^	67.28 ± 1.578^B^
Mann–Whitney (*P* value)	0.000	0.000	0.000

*∗*Mann–Whitney test: means with different manuscript letters within each column are significantly different at *P* < 0.05.

## Data Availability

The data used to support the findings of this study will be available from Dr. Mohamed Elsayed at this e-mail: elsayednada@yahoo.com for the researchers who meet the criteria for accessing this data. The data can be requested after the publication of this article. However, requests for the data, (6/12 months) after the publication of this article, will be considered by the corresponding authors.

## Abbreviations

ANOVA: Analysis of variance
WL: Working length
EDTA: Ethylenediamine tetra-acetic acid.
